# Systems biology driven software design for the research enterprise

**DOI:** 10.1186/1471-2105-9-295

**Published:** 2008-06-25

**Authors:** John Boyle, Christopher Cavnor, Sarah Killcoyne, Ilya Shmulevich

**Affiliations:** 1 Institute for Systems Biology, 1441 N 34th Street, Seattle, WA 98103, USA

## Abstract

**Background:**

In systems biology, and many other areas of research, there is a need for the interoperability of tools and data sources that were not originally designed to be integrated. Due to the interdisciplinary nature of systems biology, and its association with high throughput experimental platforms, there is an additional need to continually integrate new technologies. As scientists work in isolated groups, integration with other groups is rarely a consideration when building the required software tools.

**Results:**

We illustrate an approach, through the discussion of a purpose built software architecture, which allows disparate groups to reuse tools and access data sources in a common manner. The architecture allows for: the rapid development of distributed applications; interoperability, so it can be used by a wide variety of developers and computational biologists; development using standard tools, so that it is easy to maintain and does not require a large development effort; extensibility, so that new technologies and data types can be incorporated; and non intrusive development, insofar as researchers need not to adhere to a pre-existing object model.

**Conclusion:**

By using a relatively simple integration strategy, based upon a common identity system and dynamically discovered interoperable services, a light-weight software architecture can become the focal point through which scientists can both get access to and analyse the plethora of experimentally derived data.

## Background

Within the life sciences we continually see a steady increase in the volume and complexity of data being generated by experiments. The management of this data requires rapid development of high quality integration and analysis tools. This paper discusses a software architecture designed to fulfil this need by providing a mechanism for the structured integration of data, and analysis tools, in an extensible and straightforward manner.

Systems biology has the same data management requirements as many other fields of science, but due to its interdisciplinary nature and strong association with high throughput techniques these needs are more apparent. These requirements are to allow for: the rapid introduction of new data sources derived from new and emerging technologies; the interoperability between analysis tools written in a variety of different languages; a means to integrate data sources to support data mining and searching operations; and the ability to directly access the experimental data and associated metadata. Designs based on enterprise systems can provide solutions to these problems within an organisation, but they have to be tailored to suit the specific needs of researchers.

Life science research enterprise data integration and process management systems have evolved over the last 15 years, effectively since the creation of open interoperable object based communications (e.g. CORBA). This evolution has been from single database based solutions through to open, distributed, interoperable data management solutions. This has been driven by demands for rapid development, high levels of interoperability and increases in data volume and complexity.

**Figure 1:**
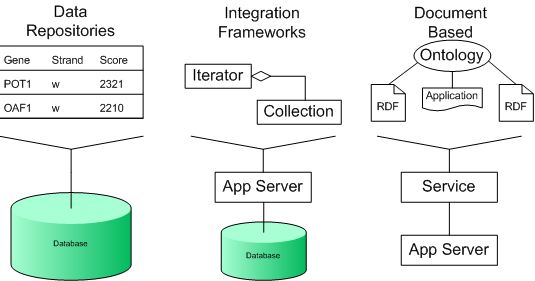
**History of integration systems for the life sciences**. Enterprise architectures for the life sciences have evolved. Limitations in the flexibility of data repositories based solutions helped shape the development of integration frameworks. Integration frameworks suffered from complexity and interoperability problems, and so document based solutions are now becoming the norm.

The development of data management systems to support the life sciences has undergone a number of fundamental changes in the last decade (see Figure 1). As in other areas, the history of enterprise systems in the life sciences is, in essence, one of a cultural change from the development of proprietary solutions, designed from the top-down, towards more flexible bottom-up architectures informed by open standards solutions. This evolution of data integration and management technologies can be categorised into three stages:

### 1. Data Centric

Initially, data repositories were developed, and integrated using either external indexing services or data warehouse mechanisms. The data repositories rely on a variety of technologies, including index based (e.g. SRS [1]), DBMS (e.g. Ensembl [2]), and federated database approaches (e.g. DiscoveryLink [3]). The development, of these data centric solutions, was driven by the availability and standardisation (e.g. OleDB, ODBC) of relational database management systems, and the requirement for a federated approach to data warehouse solutions. The lack of object interoperability of such data repositories gave an impetus for the development of top-down object centric standards.

### 2. Object Centric

With these approaches, standards bodies (e.g. LSR [4], I3C, caBIO project [5]) decided on interoperable object standards which it was hoped would be taken up by the life science industry. There has been some success with such standards, but as they had to capture all areas of a domain their complexity limited their broad-scale adoption. This object level standardisation was largely driven by the maturation of object based protocols (e.g. CORBA with pass-by-value or DCOM) and associated object services (e.g. federated registries, traders) which used interface definitions (e.g. IDL, MIDL) to formalise the static distributed interfaces. Such standards, either proprietary or open, were mainly implemented using integration frameworks built using application servers. They were introduced into a large number of pharmaceutical companies (e.g. Alliance from Synomics, GKP from Secant, Discovery Center from Netgenics). These integration frameworks suffered as their rigidly typed systems were difficult to extend and could not keep pace with evolving research requirements and new experimental technologies. Processing pipelines were also integrated within these tools. The requirements for orchestration of analysis tools led to the growth in the number of in-house tools designed specifically for rapid development and deployment characteristics, rather than interoperability or complexity (e.g. SOAPLab [6], GenePattern [7], SBEAMS [8]). The integration frameworks were built upon the maturing application server products which were principally Java based (e.g. EJB 2+)

### 3. Document Centric

Document based solutions (typically Web Service based) became popular as they provided a means to develop solutions that could: keep pace with the rapid advances in research; were scalable, robust and easy to develop; and were interoperable. These are now widely used as a basis for integration (e.g. MRC's CancerGrid, NCI's caGRID). The advantages of these approaches are based on their lightweight specifications and ease of implementation (e.g. DAS). Newer programming constructs, such as AOP, dramatically reduce the complexity of code of Web Services, and partly accounts for their widespread adoption (as can be seen in EJB 3.0, .NET framework, Spring). One of the challenges associated with Web Service architectures is their lack of semantics. In response, ontology-based solutions were developed (e.g. S-BioMoby), although these largely depended on slowly evolving globally accepted ontologies. Designs using distributed runtime type checking and type mapping are now emerging [9], as these provide for a means of integration and robustness that place fewer restrictions on developers. A number of eScience solutions were also associated with similar architectures, although the majority of these have converged with the mainstream Semantic Web (e.g. myGRID [10]). A number of tools to support these service oriented solutions have been developed, including graphical tools (e.g. Taverna [11]), schedulers (e.g. GridFlow [12]), and script translation tools (GridAnt [13]). The semantic web based solutions are being driven by both the convergence of standards for document based enterprise computing (e.g. WS-*), and the development of knowledge representations and query standards (e.g. OWL and RDF based querying solutions).

There is a natural progression with these systems, as they generally follow the traditional approaches to software designs that are prevalent at the time (e.g. MDA). This means that the designs have an inherent underlying "predilection for the pedestrian". However, with each stage the flexibility of the designs increases. This means that with directed effort it is now feasible to extend the currently available enterprise technologies to provide adaptive systems that are able to satisfy the unique nature of systems biology based scientific research. Such an approach, however, requires a new type of thinking both in terms of distributed systems and algorithm development. The traditional approaches that are currently being developed are designed to work with "finished" data, not research information. In such static views, reuse is generally based upon the development of a standardised interface, followed by adoption or implementation of the required components. This style of architecture works well within publishing scenarios, where information is to be made available across the internet as a community resource. When working within a research project, typically within an intranet, where new technologies and ideas are continually being developed, these static publishing approaches (even including semantic web and grid based technologies) are not appropriate. Instead, a flexible analysis and access system is required that allows for the rapid introduction and integration of many types of data.

This paper is technical in nature, and is designed to be of use to both software developers working within research environments and for their project managers. The results section gives an overview of why the system was developed, and describes the architecture. The discussion section outlines further areas where we feel work needs to be focused. The final section gives our conclusions about why systems similar to the one described herein are needed. A glossary of terms is then given at the end of the document.

## Results

This paper discusses the requirements and design of the ISB Informatics Infrastructure, which is abbreviated as I^3 ^(or I-cubed). I^3 ^is a light-weight service orientated architecture which is designed to integrate disparate data sources and tools without the need for significant reimplementation or modification. The system is designed to ensure that people can always get access to experiment information, without the imposition of specific formats or language restrictions. The system utilises a number of common and open standards to: provide a high of interoperability with third party solutions; ensure that the code can be easily maintained; and minimise the cost of development.

The system was designed to support communities of largely independent research groups who have a desire to ensure that their data (and tools) can be easily shared. An organisation should consider adopting this architecture, or a similar mechanism, if the following are required:

### • Non-intrusive means of integration

The approach we propose allows for tools to be integrated "as-is", without the requirement for recoding (e.g. glass box or black box integration). If it is difficult, or undesirable, to impose a high level standardisation with an organisation then such a non-intrusive development scheme will be useful.

### • Tool and language interoperability

The system is designed to support a community which uses a diverse mixture of environments (e.g. MatLab, R), programming languages (e.g. Java, Ruby) and applications (e.g. Cytoscape, Excel) by providing interoperability between both data access and analysis tools.

### • Flexible and rapid development

The software architecture, we have built, is designed to work within research environments where the needs of the scientists are continually changing. The system can be rapidly adapted to integrate new experiment technologies, analysis mechanisms and data sources.

The I^3 ^integration is designed to be "as is", meaning that wrappers (or proxies) must be implemented for each nonstandard component that is to be integrated. For standardised components generic wrappers have been developed (e.g. for JCR). The proxy systems provide the minimal descriptions of the data sources or analysis tools that are required to ensure a level of integration. For data sources the proxies must be able to map to and from a global identity scheme, so that both the data (and associated metadata) can be uniquely referenced and retrieved. Analysis tools are made available as SOAP based Web Services, with the interfaces they offer and a taxonomy defined description being published on a central registry.

This section firstly introduces the design principles we used when building the system, these were based on experiences in delivering a system that works within a multidisciplinary research organisation. The architecture of the system is then described, with an example use case and a discussion of the shortcomings of the system.

### Design Rationale

The design rationale behind the software architecture, discussed herein, has been driven by the requirements of scientists and the types of data that they generate. The architecture needed to be flexible enough to integrate research data gathered using a varied collection of technologies and platforms, including: microfluidics based cell growth analysis involving both FACS and specially built LabView controlled 'lab on a chip' platforms; proteomics mass spectrometry results generated via the Trans-Proteomics Pipeline (TPP) [14], which were generated from over 10 different mass spectrometry platforms; genomics data from both microarray expression and ChIP-chip platforms (from Agilent, Affymetrix and Nimblegen); and cellular imaging experiments involving a large number of different microscopy based platforms including confocal microscopy (both spectral scanning and spinning disk equipment), automated software controlled microscopy environments (e.g. IPLab controlled Leicas) and automated machinery (e.g. LEAP system from Cyntellect).

Flexibility is essential as the needs are research driven, so it is rarely possible to foresee the use to which data from these and future technologies will be applied. The results of these technologies have to be made available to a varied user community including: software engineers, who require programmatic access in a variety of different languages; computational biologists, who require that the results be available in a structured format for data mining and integration; and bench scientists who desire direct access to the data through a variety of tools and analyses. Therefore, the developed architecture had to be able to support a growing number of different experimental technologies and provide for a means to flexibly deliver the required data and analysis tools to a variety of environments.

To ensure that the architecture would be suitable for our needs, its design was driven by the following axioms:

#### 1. Ad-hoc development must be supported

At the cutting edge of scientific research there is a continual requirement for the introduction of new data sources, data analysis mechanisms, and changes in project focus. These requirements mean that there is minimal regard for formal design and data modelling. To ensure the expediency of software, it is typically continually being produced through rapid development in an isolated and non-formally designed manner (e.g. through the use of mathematical functional languages using tools such as Matlab or R). This lack of structured (formal) design is part of a growing global trend [15]. This trend is exasperated by the fact that within the scientific community there exists a rich and varied user base, which ranges both in terms of software experience (typically including lab scientists using macro languages, statisticians and computational biologists using script based tools, and software engineers developing enterprise solutions) and the plethora of computing languages they use. This means that it is important to accept that ad-hoc development is the norm, and that scientists are required to work in this manner if they are to carry out their research effectively. To avoid a continuing series of iterations of extensive and expensive reimplementation, strategies must be developed to support such development practices. To support integration tasks requires either: the development of a post hoc integration strategy (e.g. a means to migrate scripts to a production environment); or the provision of a simplified integration mechanisms, which the developers can use conveniently (e.g. using directly resolvable identifiers/URNs, so that data items can always be mapped to each other and any associated metadata).

#### 2. Documents, not objects, are more useful to scientists

Over the last decade, in distributed computing, there has been a shift in philosophy from thinking in terms of the *transportation of object graphs *towards the *retrieval of related documents*. The distinction between documents and objects is subtle, albeit important: objects are for programs, whilst documents are for people. An object is by its very essence a "black box" which contains domain and platform specific information. Objects must be explicitly translated between languages, and must be serialized (marshalled/externalised) for transmission through an object protocol (e.g. CORBA, RMI, DCOM, .NET Remoting). Documents are open and readable, and so lend themselves more easily towards the social aspects of a distributed system. With a document centric approach the interactions are more natural and flexible: the document can be saved and retrieved from a file system using standard desktop tools; the information can be retrieved through numerous media, for example through a web page or from an email received from a colleague; and documents can be directly browsed and their contents edited. This shift in thinking has been driven by changes in technology, in essence: we now have the desktop computing power required to deal with operations associated with handling and transforming large numbers of documents. The detachment of documents from the underlying storage does present a challenge when supporting collaborative operations, as generally concurrent and transactional control strategies have to be implemented that work within a stateless environment.

#### 3. Scientific enterprises operate better through a bottom-up service oriented, rather than top-down application oriented, architecture

As discussed above, the architectural decisions made for systems biology based research enterprises are driven by the need for functionality and rapid development rather than shared data models and unit tests. The traditional top-down approach to design is rarely used, with a bottom-up approach being prevalent and arguably preferred [16]. In the top-down approach models are defined a priori and the designer uses them as the 'lingua franca'. By contrast, in the bottom-up approach models and designs arise out of ad-hoc development and are user driven. To ensure that software can be maintained, and that it is possible to build upon the work of others, a combination of top-down design with bottom-up development practices is often needed (this is referred to as 'middle-out'). With 'middle-out' systems, top-down models are present, but are not tightly bound to the individual data stores. Middle-out solutions generally work through either the use of high levels of abstraction which can be easily used by 'bottom-up' developers (e.g. relationships, life cycle, identity services), or by allowing for formalised descriptions of data and tools to be decoupled from the implementation (e.g. BioMoby [17]).

#### 4. Adoption and adaptation of technologies is better than "reinventing the wheel"

The risk with any research lead computing project is that the novelty (or originality) of the software can become more important than the production of innovative functionality required to meet the demands of science. By focusing on innovating through using and extending existing solutions, the best use of the available computing technologies can be made. In the past a number of high profile "omics" integration projects have initially ignored the current mainstream software architecture and standardisation efforts. Within a few years these larger projects (e.g. caBIG from NIH, myGRID from the UK eScience initiative) have aligned themselves back with the mainstream. An example of such adoption is the migration to standard technologies such as Web Services and Hibernate by later versions of the caCORE infrastructure. The reason for this realignment is that these large scale projects have found themselves attempting to implement solutions which are clones of already existing well maintained generic systems. These projects have gone on to innovate, but they have done so by building on top of existing standards and solutions. When building systems it is essential to reuse the best tools from other application areas as this ensures a high return on investment. This high return results directly from not continually developing *de novo *solutions, which is a waste of resources and funding, and typically leads to a sub standard and non maintained solution. By using and expanding available computing technologies, the community can also gain an understanding as to their limitations and what is required to ensure that they can become more appropriate for research.

These axioms led us to develop the solution described in the next section, which: is based on distributed documents, allows for a high level of interoperability and multiple integration mechanisms; uses third party components, so that there is a high level of standardisation and the development costs are kept low; and does not impose a rigid highly formal software structure, thus allowing the scientists to keep the flexibility they required to undertake research.

### Distributed Architecture

**Figure 2:**
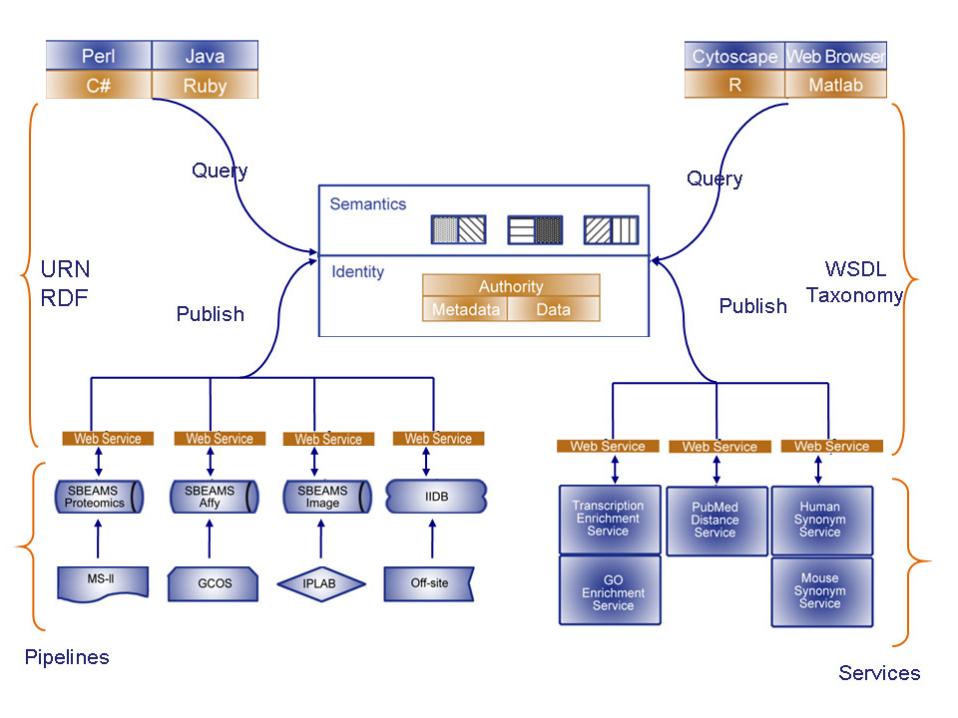
**Overview of I^3 ^architecture**. The system is loosely coupled and identity driven, so that services and data are dynamically discovered. There are two sides to the architecture: data access and data analysis. Data access uses an identity system for mapping data items to each other and to their metadata. Data analysis is based around Web Services, with descriptions of the services being stored in a registry service, so that resources can be reasoned over and discovered at run time.

The I^3 ^is a modular, service-oriented, research enterprise architecture which is capable of integrating emerging technologies (see Figure 2). This enterprise architecture is designed for interoperability and extensibility, and uses the facets of both 'top-down' and 'bottom-up' design (i.e. middle-out). In I^3 ^developers can use their own evolving data models (bottom-up). However, formally defined domain specific data models and services (top-down) are provided through a number of services and formal definitions.

This architecture is designed to be flexible, interoperable and light weight, while enabling the rapid development of new solutions and integration of new technologies.

The solution discussed in this paper uses two approaches for providing the desired middle-out functionality:

• A central registry is used to define the functionality of distributed data analysis services. The data analysis architecture is based around Web Services, with an ontology describing the Web Service being stored in a registry service, so that resources can be reasoned over and discovered at run time. A central registry service is used to store information about a set of individual services, including: data about the functionality of the service; and the group which offers the services. This means that the formal top-down descriptions are detached from the underlying bottom-up services. These descriptions are standardised through the use of a taxonomy, which describes the analysis methods and the data that the registered services provide. This "separation of concerns" means that the services can be written easily and the definitions can be updated independently of the individual services. Such approaches do have limitations in terms of complexity of data/algorithms that can be described, and do require good coordination to function. A number of alternative specifications which follow this pattern have been proposed [17].

• A unifying identity based system is used for data access. The data access uses URNs, which are encoded as Life Science Identifiers (LSIDs) [18], to provide an identity system for mapping data items to each other and to their RDF defined metadata (relationships between data items are also encoded using LSIDs). This strong unifying identity system is used to link low level data-oriented bottom-up services into a structured top-down infrastructure. The LSID identity system has the advantage of being namespace based, so allowing for the integration of a number of independent identity schemes. With a unified identity scheme each item can be referenced, and so structure can be imposed through the use of either: additional services (e.g. relationship services to provide information about links between items); the use of a project system (e.g. to store information about collections of items and annotations); or through the association of metadata as structured information (e.g. RDF). A number of alternative ID schemes are also available (e.g. UUID based, or PURLs).

Alternative middle-out solutions are becoming available. In particular, the semantic web (Web 3.0) provides a means to develop middle-out solutions, with each service being closely coupled with a formalised description. The semantic web offers a range of technologies which are useful (e.g. OWL for definitions and SPARQL for access/querying), although this work is still ongoing. The advantage of the Web 3.0 approach is that data and semantics can be served out using the same mechanism.

The I^3 ^does not directly impose a structured data model on clients, the system uses a standardised ID mechanism coupled with the use of 'meta models' and service ontologies. This means that to integrate new data sources "proxy components" must be constructed which can provide mappings between underlying domain specific identities and the universal identity system. In this way, all data items (and their associated metadata) are uniquely identifiable and can be directly retrievable. To ensure a high level of interoperability, the integrated tools are made available through SOAP based Web Services. All the tools and data sources are registered with a central registry system, so that they can be discovered at runtime.

The key features of the system are:

#### • Interoperable

The system is designed to be interoperable between the target programming environments (e.g. Java, Ruby, Perl and C#). Interoperability tests were developed for each of these languages, and the services within the system adhere to a subset of the WSDL1.1 document/literal standard. These provide a collection of standards for making remote method calls to retrieve and analyse information. In brief, the salient Web Service related technologies are: SOAP which provides the means for transporting documents to and from a Web Service; the WSDL which defines the interface for the Web Service; and the UDDI registry which stores information about the location and features of the Web Service. These technologies have undergone a number of evolutions, and with the adoption of the WS-I basic profile there is now true interoperability between Web Services. The use of standard *literal documents *coupled with a well defined interface (e.g. SOAP 1.2 with WSDL1.1+) means that clients and servers from the major languages can now interoperate in a stateless manner. The WS-* standards are a move towards a level of interoperability which will provide the features that are necessary to support a true enterprise application. By providing a high level of interoperability, researchers can develop algorithms in the way they prefer.

#### • Rapid Application Development

An identified problem with SOAs is that their lack of formalisation means that their adoption within an organisation is problematic. The problems that arise are due to the fact that people can use the services without having to integrate their code. This means that widespread adoption of the architecture requires political persuasion, as well as technical argument. To encourage adoption rapid application development (RAD) is needed. RAD requires both rapid integration of services by clients and rapid deployment of services into the architecture. The architecture supports rapid deployment of services by: the use of 3^rd ^party code for the generation of LSID services and development of Java based Web Services from skeleton code (using Axis 2 from Apache); and the rapid development of R-based Web Services (using a purpose built Ruby proxy tool which allows for the semi-automatic writing of marshalling code and WSDL definitions). Rapid integration for the client is supported as developers only need to know how to access their data (through automatically generated WSDL defined stubs) and are not required to adhere to a pre-specified object model.

#### • Non-intrusive

The system is designed to be non-intrusive, so that different groups can use their own object models and data formats. As there is no overriding object model, developers can integrate with the architecture in a post-hoc manner. Fundamental to such non-intrusive development is the use of a hierarchical identity system, which can be used in the 'bottom-up' style of SOAs that arise within the research enterprise. By using such an identity system, each group can develop their own sets of identity assignment and resolution services, which can be integrated with external resources. We have used the hierarchical naming system LSIDs, which provide a unique ID system based on URNs, where a unique identifier is sent to a server which dynamically resolves it to a data document and metadata. The metadata is encoded as Resource Description Framework (RDF) documents. As the identifiers themselves are resolved, the client can always drill down to the experiment information without having to conform to a specific object model or data format.

#### • Extensibility and Maintenance

Wherever possible we have used common data and computing standards. This means that we benefit from using the best of 3^rd ^party applications (e.g. jUDDI, Apache SOAP, LSID stacks, JCR, common ontologies). Such use of 3^rd ^party tools means that both the development and maintenance costs are considerably less than with *de novo *development. The system is designed to be dynamically extensible, so that new services can be integrated using standard protocols and tools.

#### • Dynamic Discovery

Central to the system is a registry service, which can be reasoned over to discover the required service(s) at run time. While Semantic Web technologies are not yet mainstream, aspects of their behaviour can still be used within any distributed environment. We have defined ontologies to describe both the data and the services that produce them. The service ontology is three-faceted, and describes different aspects of the services: an administrative ontology is used to define behaviour associated with maintenance and versioning of the service; a functional ontology is used to provide a high level description of the group who control the service and the function to which it is applied; and a descriptive ontology allows for the development of textual tags. The service description is stored in the registry, so that it is detached from the service itself.

We have used I^3 ^to support a number of active research areas at the ISB, including genomics, microfluidics and imaging. An illustrative scientific application which has been built using this architecture is given in the *example high throughput imaging section *below. Further information about technologies and usage are given on the associated web site [19].

### Example: High throughput imaging

We have applied the infrastructure in a number of core areas at the ISB. In particular we have used the system to develop: high throughput technology data repositories (e.g. genomics, proteomics and imaging) and distributed analysis systems (e.g. distributed 'R' language analysis tools, imaging analysis pipelines, array annotation pipelines).

**Figure 3:**
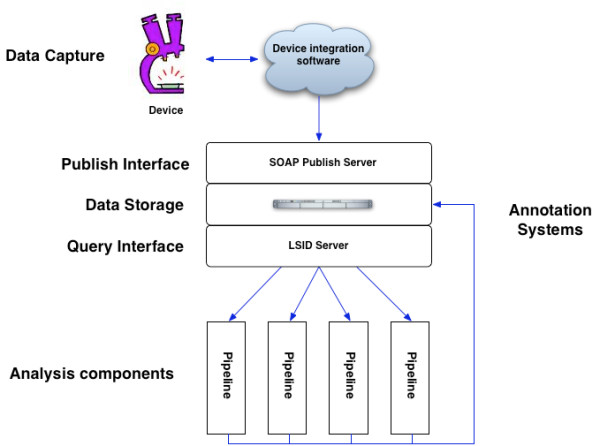
**Example usage of architecture for high throughput imaging**. We have used I^3 ^to provide a uniform mechanism for the capture of information from and the control of this instrumentation. For microscopy based imaging an end to end data capture, and control, system has been implemented. Image data is captured directly from the microscopes and specially built drivers are used to integrate the equipment. The data is captured from the device, parsed into an intermediate form and published via a SOAP interface to a data store. The data is held in a staging area in the data store until resources are available for processing, once processed the data can be queried via both LSIDs and SOAP.

An area where we have applied I^3 ^is in the development of software for the automatic analysis of high throughput cellular imaging (see Figure 3). The imaging system consists of a number of services, each of which is dynamically locatable through our registry service. As these services are designed to be orchestrated externally, they can be reused within other distributed applications. The image data is captured directly from the microscopes and specially built drivers are used to integrate the equipment. Access to the image repository service is through a SOAP publish interface. When the data and associated metadata are published they are passed through an extract-transform-load (ETL) system into a data repository. The ETL system consists of a staging area and a resource managed state machine. Once the experiment information is available in the image repository service it can be browsed and queried using two different mechanisms: a SOAP/REST based interface is available to provide querying and searching functionality; and an LSID endpoint is available to provide direct retrieval of the data and associated RDF encoded metadata. Analysis modules are written against these query interfaces, and are run from within a specially built analysis service (which is underpinned by GenePattern). The system has been designed to scale to the level of throughput required by the current generation of cell population based imaging experiments.

By integrating the repository with I^3 ^we are able to provide interoperable access and retrieval of the imaging information. Such integration involved the development of a data access component and the development of Web Services for publishing and querying. Explicitly development of the data access system required:

#### • Document Demarcation

The granularity of the information that is to be served out, and the relationships between these items, must be specified. In this case the documents mirror the internal repository structure, which in turn mirrors the OME [20] specification: project documents describe a project (e.g. owner and description) and provides URNs for related experiments; experiments describe the conditions and equipment for individual microscopy runs, and provides URNs for the captured images; and image (and image set) documents describe the individual images, including extracted features and image characteristics.

#### • Define the RDF metadata

The metadata associated with each data item must be formalised. We use Dublin Core for describing relationships and basic document parts. Domain specific ontologies are used to describe specific types of experimental metadata.

#### • Build the endpoint

The LSID specification allows for FTP, HTTP and SOAP endpoints. In this instance a SOAP endpoint was used. This endpoint was constructed using the freeware LSID stack. Alternatively the endpoint can be constructed against the standardised WSDL and deployed under Apache Tomcat.

#### • Registration

The namespace for the image repository, and the endpoint it should resolve to, were then registered with the ISB central authority.

Image analysis was undertaken utilising GenePattern, and so a query and publish interface were developed to allow for individual analysis pipelines to pull information from the repository, and for uploading of information. To ensure the interoperability and integration with other tools these were implemented as Web Services by:

#### • Publishing and Query interface definition

The WSDLs were developed following a specific subset of the document/literal WSDL 1.1 specification. This was done to ensure interoperability with our main client platforms. The services themselves were built using Apache Axis, and deployed under Apache Tomcat.

#### • Registration

The services were registered with the central service registry, so that they could dynamically discovered This involved adding information about the endpoints, administrative information (including version and group information), and information about the type of data/area within which the services function.

### Shortcomings of I3

All middle-out solutions have limitations, as they attempt to overlay structure on ad-hoc and unstructured services. Generally middle-out solutions sacrifice richness of functionality to provide flexible systems. The requirement for interoperability and the reliance on standardised solutions result in a number of disadvantages with the adoption of the architecture discussed in this paper. The major drawbacks to such a loosely coupled middle-out solution are:

#### • It is Stateless

The main mechanisms for associating state information, through external service(s) or use of a *current *thread mapping system, are not applicable to the protocol independent document based interoperability mechanism used within our system. As the method calls are inherently stateless, custom engineering or a stateful specification (e.g. session management calls) are required to introduce state within the system. With the convergence of the relevant standard implementations (e.g. WS-RF family) this problem can be addressed, although true interoperability will require further maturation of this family of technologies.

#### • Security is single logon

The system relies on the underlying protocol to provide security (e.g. SSL session based), which means that fine level security based on an access control list (ACL), or similar, is not available. As with state behaviour, there are a number of standards that will provide this functionality in the future, but no truly interoperable implementations are currently available.

#### • Concurrency and Transactional behaviour are not supported

There is no simple mechanism to support resource locking or leasing. This lack of distributed transaction (e.g. two phase) behaviour will cause problems with multi-step analyses, typically through the occurrence of lost updates. Again, while this problem arises due to the lack of current thread identity or explicit token transference, there are solutions based on either custom engineering or arising standards efforts.

#### • Not process oriented

Top-down defined behaviour, typically in the form of workflows, is an essential part of scientific analysis. Such process-oriented architectures generally require top-down design, as failsafe behaviour is generally essential. While the architecture does not directly support this type of processing, workflow systems could be overlaid on the services to provide process-oriented views (e.g. through BPEL scripts). While such scripting will allow for distributed processing, there is still a disadvantage with using the architecture for high demand large scale processing, where the most efficient solution is to move the analysis to the data (and not vice-versa).

While none of these disadvantages are *showstoppers*, it is important to recognise that such ad-hoc service oriented architectures have limitations. These limitations can be overcome, but they require custom engineering which is strongly dependent on the individual enterprise.

## Discussion

It is no surprise that the evolution of enterprise software for the life sciences closely follows technological and methodological trends. There is an implicit understanding that there exists a commonality between all enterprise systems, in terms of the underlying design. It is assumed that information and processes are the same no matter what the domain. Unfortunately, this perceived commonality is a fallacy when applied to the life sciences. Software is only part of the solution and the way in which people work and interact is equally important and cannot always be captured using simplistic requirements gathering methodologies. In complex computing endeavours, the social demands go beyond those usually considered in more traditional "factory software" design and development.

There is no such thing as the quintessential scientific research project, as every one is unique. These differences arise as each investigator and investigation studies the unknown, which means that the rationalization, which is typically needed for the design of expedient software utilities, is missing. However, it is possible to develop a level of rationalization about such studies using abstractions which describe different facets of the data and process (e.g. data can be captured, equipment can be controlled, content can be managed and analyses can be undertaken). The majority of "off the shelf" scientific information integration systems are generally targeted towards the end results of research, rather than aiding in the progression of scientific understanding. To support the needs of research driven science, architectures need to be provided which can be adapted to new usage and have a low maintenance cost. This flexibility requires working at a level of abstraction that is atypical for most software projects. To build systems which others can readily use we need to focus on the fundamentals of content and identity, rather than solely attempting to model biological entities. Fortunately, there are a number of existing technologies which can be used as the foundations for such endeavours (e.g. content repositories, identity driven retrieval systems).

Some of the development effort in a research environment is for one-off applications, while other projects are geared towards more general usage. With general usage software, even if it is well written with good interfaces, the chances of the code surviving much past the end of the research funding is small. The problem is largely one of adoption and usage; unless there is a demand for the code and it can easily be adapted for new usage, it is impractical for other groups to adopt it. This is not a new phenomenon in software engineering [21]. That stated, there are areas where general software utilities can be developed and are needed:

### • Design of cross-cutting services

We recognise that the need for a "systems biology enterprise" is to develop an architecture that provides tools for manipulating data, without necessarily understanding all aspects of the contents. While information is an abstract concept, it is possible to formalise how we deal with it within the enterprise. This formalisation, in a service oriented architecture, takes the form of generic cross-cutting services which can be used in a large number of varied applications. The Object Management Group (OMG), and other standards bodies, have outlined the functionality of such services, including: relationship service, which can dynamically provide relations between documents; a synonym service to find documents of terms that identify the same concept; a query service to search through collections; a registry service to host information about other services; and a lexical service to allow for a common ontology to be used across different applications. While implementations of these services exist, few are generic or mature enough to be readily available. We feel that the development and maintenance of such horizontal services will be of benefit to the systems biology community, and so a large proportion of our future work will be in this area.

### • Development of services to support top-down analysis

There will always be a need for structured top-down formalised analysis of scientific information. To allow for such an analysis, middle-out architectures, which marry the benefits of bottom-up and top-down, are an ideal solution. Such middle-out architectures can be achieved through the use of structured documents, as we have used in I^3^. Additionally, the adoption of technologies that will allow for the overlay of top-down functionality on a standardised SOA can provide the required functionality. In particular, the Business Process Execution Language (BPEL) and associated graphical tools offer an attractive and low cost mechanism to provide a means for the rapid development of top-down workflows.

### • Dynamic data resolution systems

Dynamic discovery of information is essential in all data driven domains. The use of formalised methods to ensure the resolution of individual data items, and relationships between these data items, is essential. As systems biology is largely data driven, we advocate the adoption of a dynamic hierarchical identity system (e.g. URNs encoded as LSID or similar). A hierarchical identity system allows each group, with an organisation, to use their own namespace and ensures that that no matter what format the data is in, or the delivery systems that is used, it is always possible to resolve back to the original experimental results. We also use structured documents (RDF) to describe metadata, these can also be used to capture a wide range of relationship types. The modelling of these relationships typically evolved past the traditional composition (part of), aggregation (has a) and generalisation (is a) relationships towards operational relationships which support a richer semantics based on both our scientific understanding (e.g. spatial, temporal and logical) and the data provenance. Any data resolution system will be expected to understand data provenance information including: history information to allow for the discovery of different versions of documents; view information to allow for the discovery of context dependent transformed documents; and query information to allow for the dynamic discovery of related documents. At present there are no readily available implementations of an identity scheme that support all the required functionality.

## Conclusion

The coupling of rapid development (e.g. using mathematical scripting tools and functional languages) and rapid deployment (e.g. through Web Services) means that it is now relatively easy for disparate groups, both within the enterprise or throughout the Internet, to share information in an *ad hoc *manner. This trend is even more apparent at the cutting edge of scientific research in systems biology, where there is a continual requirement for the introduction of new data sources, data analysis mechanisms, and changes in project focus, which means that formal design and data modelling are minimal. Within the scientific community there exists a rich and varied user base, some develop software and some require a reliable analysis system. The development community ranges both in terms of software experience and the languages they use. This paper perpetuates the view that the successful development of an architecture to support such research requires a different strategy to that of mainstream enterprise systems. The adoption of grid systems or Service Oriented Architectures are only a first step towards the next generation of enterprise systems that will be of use to the scientific community. We feel that due to the rapidly evolving requirements of biological research, when compared to that of rigorous software development, the next generation of software architectures will be required to support bottom-up integration. This means that architectures will have to work at a higher level of abstraction, so that they can be used to integrate data and tools without the need for costly reimplementation. The core of such integration will be operations using a rich identity driven data system coupled with domain specific descriptions.

The development of integration strategies for systems biology is problematic due to both the nature of science and the organisation of scientists. It is typical that the means to which a specific technology will be used, and the methods used to analyse the resulting data, is unknown at software design time. Scientific understanding evolves, and so too does the work that is undertaken. A software architecture that is designed to aid in such endeavours has to be designed to support such evolution, meaning that traditional approaches to development can rarely be applied. As we do not know how the data will be used or analysed, a flexible solution, like the one discussed in this paper, is needed. In the future, we can expect a growth in the requirement for such unstructured development. Thus, the supporting integration systems will need to provide for the flexible post-hoc integration of black box components as a fundamental design principle.

## Availability

Related downloads and documentation can be found at the ISB informatics group page: 

## Abbreviations

***BPEL***. The business process execution language is a specification designed to support the high level orchestration of web services. The heart of the BPEL specification is the scripting language which defines how services and data produced by them are linked together. This specification is rich enough to allow for most workflows and defines both how method invocations and data are linked and the how web services should be coordinated (e.g. concurrency, choices, sequential operations). The specification also defines extensions to the WSDL which can be used to specify links between services (which can be discovered dynamically through UDDI querying). A number of implementations are available, including those that run under JBOSS [22] and ODE from Apache [23]. Information about the specification can be found at OASIS.

***CORBA***. The Common Object Request Broker Architecture supports interoperability between distributed processes (applications). Central to the architecture is an ORB (object request broker) which both marshals data and controls compartmentalisation (to allow for invocation on specific the threads etc) of between the different processes. The specification was defined by the OMG, and ORBS are available for most platforms.

***DAS***. The Distributed Annotation System [24] defines a protocol for the retrieval of annotations from genomes. Requests are sent using http encoding which returns an XML document. A number of clients and servers are available, and it is used in a number of large scale genome projects (e.g. Ensembl).

***DBMS***. A database management system is the environment in which database instances exists. A DBMS provides a unified framework which can be used to control the physical (tablespaces), conceptual (logical schemas) and external (views) of databases.

***DCOM***. Provides for a means to make distributed calls between COM (Component Object Model) objects. Thread compartmentalisation and marshalling (using low level XML interchange) is handled automatically. For application developers this has largely been superseded by .NET Remoting.

***EJB***. An Enterprise Java Bean (EJB) is a server side component that lives inside an EJB Container. There are different types of EJBs, and each has a different purpose: an *entity bean *which serves as a data cache from an underlying data store, this is used for transformation and data integration logic; a *session bean *which is typically used to hold application logic which communicates with information stored within entity beans; a *stateless session bean *which typically represent simple stateless logic and generally act as end points for high level services; and a *message bean *which is used to pass message between the other beans. The EJB 3.0 standard (2006) represented a major change as the complexities of developing EJBs was inhibitive for most projects, so a simplified process of building EJBs was outlined (through the use of detachment, dependency injection and aspects). Full details are available from Sun [25].

***EJB Container***. The EJB container controls the life cycle of the bean, facilitates access to core services and manages server-based resources. The services that are available through a EJB Container provide: security management, including method level and ACL; transaction management; including 2 phase/distributed commits; life cycle management, including pooling of bean instances and swapping beans in/out (persisting) of memory for resource management; naming/directory management, typically through JNDI; persistence management; using ORM tools such as hibernate; remote access management, so that the bean can be accessed via RMI-IIOP/CORBA and Web Services; and a number of utility services (e.g. mailing, clustering, caching, monitoring). A widely used, and free, container is JBOSS [26].

***I3C***. The I3C was a short lived commercially led organisation established to standardise aspects of life science informatics. The organisation was led by Oracle, Sun and IBM. The I3C did promote the use of LSIDs, which have been adopted by the OMG.

***IDL***. The Interface Definition Language formalises the remote interfaces that can be accessed through CORBA. IDL has evolved considerably, with the advent of pass-by-value and components (facets). A WSDL serves the same type of purpose for Web Services.

***JCR***. The Java Content Repository is a specific Java Standard (JSR-170) for defining the interface to a content repository. A content repository is a flexible system that is typically customised for a specific usage, when customised it is referred to as a Content Management System (CMS). A number of implementations are available, including Jackrabbit which is licensed through Apache [27].

***LSID***. The Life Science Identifier standard [28] provides a concrete definition and implementation of a URN. The LSID specification outlines how the URN is resolved to two locations (the data and the metadata) through the use of "an authority". In this way the authority acts as a registry. The documents that are retrieved are returned as objects and an associated RDF data file which encodes the metadata. The standard also encompasses many aspects of using URNS, and includes specifications for associated services (e.g. assignment). Details about the specification and implementations are available [29].

***LSR***. The Life Science Research group of the OMG [4] defines standard in the "vertical" life science domain. The body have defined and adopted a number of standards. These standards cover a wide range of areas (including "sequence" and "literature").

***MDA***. A Model Driven Architecture is one where the model underlying the system is defined in a language independent way, and the corresponding services/classes are automatically pushed out from that model. Typically the model is defined in UML and them XMI is used to automatically generate stubs/skeletons which can be used to provide implementations of the model.

***MIDL***. The Microsoft Interface Definition Language serves a similar purpose to IDL, but is generally based on specifying the remote procedure call interface which is used between COM components.

***.NET Remoting***. The .NET framework provides a mechanism for making remote calls called Remoting. Remoting includes many useful features for the development of distributed systems, these include: life cycle management, so that distributed behaviour/GC and leasing can be controlled; protocol support for binary socket based communication and other streams; and specification of the behaviour of a remote service/object (e.g. singleton).

***ODBC/OLEDB***. The Open Database Base Connectivity is a definition of the interface presented by a DBMS. The ODBC specification is well established and bridges with other technologies (including JDBC). The OLEDB is an extension to the ODBC offer richer functionality.

***OMG***. The Object Management Group [30] is an open not for profit standardisation body. The OMG have produced a number of horizontal (e.g. Trader service, Naming service, Event Service) and vertical (see LSR [4]) standards for use with CORBA.

***OWL***. The Web Ontology Language is an RDF description of an underlying data resource. The ontology describes the data items produced through a web service as well as the relationships between them. Details are available from the W3C [31].

***RDF***. The Resource Description Framework is a W3C (WWW consortium) standard for describing resources available on the Web. RDF forms the basis of formalised descriptions of services (e.g. OWL) and can be used in conjunction with extensible metadata descriptions (e.g. Dublin core [32]). RDF consists of a series of connected triples, so that complex representations can be constructed as a graph. Details are available from the W3C [33].

***REST***. Representational State Transfer (REST) [34] can be considered an alternative to SOAP, although it is considerably easier to implement. REST uses pre-existing technologies as the basis for the protocol (e.g. "the web is the platform"). There exists some confusion about what represents a Restful service, rather than just an HTTP encoded request for an XML document. True REST is based upon the verb/noun/type based calls, where you apply an operation (verb e.g. POST, GET, PUT and DELETE) to a URI (noun) with a certain view (type).

***RMI***. Remote Method Innovation is a Java-to-Java solution for communication between distributed Java threads/applications. RMI uses a number of abstraction layers (remote reference layer/RRL and transport layer), this has a number of advantages including the fact that different underlying protocols can be used to actually provide the communication (e.g. IIOP). Marshalling is done through serialization, leasing is available, and distributed GC is supported. RMI is a convenient, but not interoperable, protocol.

***SOA***. A Service Oriented Architecture is one which consists of loosely coupled federated services. There is typically little linkage between these services, and they are generally discovered dynamically using a registry system or similar. SOAs have grown in popularity within many enterprises, as they provide a practical mechanism for disparate groups to share information/processes.

***SOAP***. SOAP is a protocol for making requests on remote services to return structured data. It is designed to use a any high level protocol that supports the sending of information, and is primarily used with HTTP. Much like CORBA, interoperability is the big draw of SOAP, and (unlike CORBA) SOAP has the advantage of being simple to develop and test. The original stateless nature of SOAP limited it usage, however with the advent of WS-RF (and other standards) SOAP is maturing into a general purpose object protocol. More information about SOAP specifications is available from the W3C [35].

***SPARQL***. The SPARQL Protocol and RDF Query Language is designed to allow for the querying and retrieval of documents across multiple unstructured data stores. The power of the system is the distributed RDF documents (or other data stores) remain unchanged, but queries can be run across them – and so it fits well with a "bottom-up" approach. Such a unified approach to accessing information is required to make the semantic web (Web 3.0) a reality, and there do already exist some implementations (e.g. Virtuoso [36]). More information about the query specification is available from the W3C [37].

***UDDI***. Universal Description Discovery and Integration is a WSDL (therefore interoperable) defined registry/dictionary system. UDDI 2.0 is the currently used version, and it supports the registration and querying of Web Services using specific mappings. OASIS [38] have details of the different standards (version 2 and 3), and Apache have jUDDI which is a 2.0 implementation [39].

***URN***. A Uniform Resource Name is a type of URI (Uniform Resource Identifier). It is the logical counterpart to a URL, in that it provides the name of a resource rather than the exact location of a resource. A number of URN implementations are available, including LSIDs.

***Web Services***. A Web Service is a server which performs request/response operations and (generally) works using documents. A request is sent (either as a well formed document or using http encoding), and a well formed document is returned. The term Web Service originates from the fact the web based protocols are generally used to provide the communications.

WS-*. The WS-* are a series of specifications for adding functionality to SOAP. These extensions provide new functionality such as security, messaging, binary object attachment and state. These extensions generally involve the addition of information to the SOAP message (within the envelope). State information can be maintained between SOAP calls through the use of resource frameworks (e.g. WS-RF). OASIS [38] keep a large number of specifications.

***WSDL***. The Web Service Description Language provides a means to specify the interface exposed by a SOAP Web Service. The WSDL document can be automatically retrieved, and tools can be use to generate convenience classes for specific languages, so that no XML parsing code needs to be written by the developer. When writing a WSDL a number of standards (e.g. WS-I) are available to ensure interoperability, typically though the use of profiles with literal/document "styles". The W3C have details of the standard [40].

## Authors' contributions

JB designed the system, managed the development team, and drafted the manuscript. SK worked on the high throughput imaging system, worked on key services and contributed to the manuscript. CC contributed to the manuscript, provided implementations, and standardised the metadata for the services. IS instigated and guided the project. All authors read and approved the manuscript.
